# Novel *MFSD8* Variants in a Chinese Family with Nonsyndromic Macular Dystrophy

**DOI:** 10.1155/2021/6684045

**Published:** 2021-08-17

**Authors:** Qin Xiang, Yanna Cao, Hongbo Xu, Zhijian Yang, Liang Tang, Ju Xiang, Jianming Li, Hao Deng, Lamei Yuan

**Affiliations:** ^1^Department of Basic Biology, Changsha Medical University, Changsha, China; ^2^Center for Neuroscience and Behavior, Changsha Medical University, Changsha, China; ^3^Academics Working Station, Changsha Medical University, Changsha, China; ^4^Department of Ophthalmology, The Third Xiangya Hospital, Central South University, Changsha, China; ^5^Center for Experimental Medicine, The Third Xiangya Hospital, Central South University, Changsha, China; ^6^Department of Rehabilitation, Xiangya Boai Rehabilitation Hospital, Changsha, China

## Abstract

**Purpose:**

To identify the molecular etiology of a Chinese family with nonsyndromic macular dystrophy.

**Methods:**

Ophthalmic examinations were performed, and genomic DNA was extracted from available family members. Whole exome sequencing of two members (the proband and her unaffected mother) and Sanger sequencing in available family members were performed to screen potential pathogenic variants.

**Results:**

Novel compound heterozygous variants, c.1066C>T (p.Pro356Ser) and c.1102+2T>C, in the major facilitator superfamily domain containing 8 gene (*MFSD8*) were suspected to be involved in this family's macular dystrophy phenotype. The novel c.1066C>T variant in the *MFSD8* gene probably resulted in substitution of serine for proline at the 356th residue and was predicted to be “uncertain significance” through *in silico* analyses. The novel c.1102+2T>C variant in the *MFSD8* gene was likely to affect the splicing form and predicted to be “pathogenic.”

**Conclusion:**

The novel compound heterozygous variants, c.1066C>T (p.Pro356Ser) and c.1102+2T>C, in the *MFSD8* gene are likely responsible for the isolated macular dystrophy phenotype in this family. This study enlarged the *MFSD8* gene mutant spectrum and might provide more accurate genetic counseling for this family.

## 1. Introduction

Inherited retinal dystrophies (IRDs) are comprised of a genetically heterogeneous group of ocular disorders with a wide clinical phenotypic spectrum, which have an approximated incidence of 1/3000-1/2000 [1, 2]. Inherited macular dystrophies are clinical subtypes of IRDs, which are featured by bilateral central visual impairment, macula atrophy, and underlying retinal pigment epithelium degeneration [3, 4]. Though the age of onset is variable, it often occurs in the first two decades of life [5]. Most of the diseases are rare and have common visual impairment and usually only show mild initial fundus alterations [3]. Autosomal dominant, autosomal recessive, X-linked recessive, and mitochondrial inheritance patterns have all been described in macular dystrophies [5]. In more than 270 disease-causing genes involved in IRDs, mutations in at least 32 genes including the major facilitator superfamily domain containing 8 gene (*MFSD8*, OMIM 611124) have been described to be associated with inherited macular dystrophies (the Retinal Information Network, RetNet, https://sph.uth.edu/Retnet/, last updated on January 21, 2021).

The *MFSD8* gene, spanning approximately 48 kb and 13 exons on chromosome 4q28.2, encodes an MFSD8 protein with 518 amino acids [6]. The MFSD8 protein is a transmembrane lysosomal protein that possesses two N-glycosylation consensus sites, twelve membrane-spanning domains, and the cytosolic N- and C-terminus [7]. Recently, compound heterozygous *MFSD8* variants, p.Glu336Gln/c.1102G>C and p.Glu336Gln/p.Glu381^*∗*^, have been reported as being associated with nonsyndromic macular dystrophy [8].

Whole exome sequencing has recently been successfully applied to reveal the genetic causes of hereditary ocular diseases, especially disorders that are extremely genetically heterogeneous, such as IRDs [9, 10]. In this study, novel compound heterozygous variants, c.1066C>T (p.Pro356Ser) and c.1102+2T>C, in the *MFSD8* gene were identified as the potential genetic cause of this Chinese family with nonsyndromic macular dystrophy by whole exome sequencing and Sanger sequencing.

## 2. Subjects and Methods

### 2.1. Subjects and Clinical Assessment

**Figure 1:**
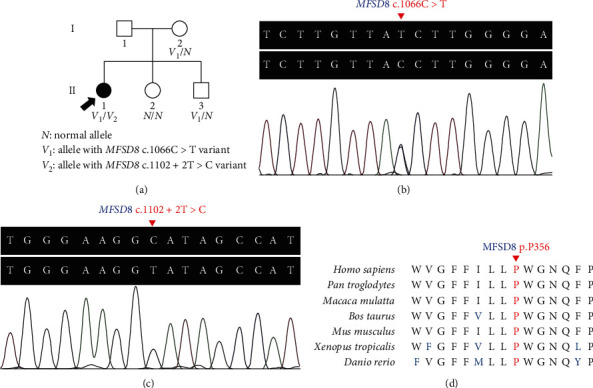
Compound heterozygous *MFSD8* gene variants identified in a macular dystrophy family. (a) A Chinese macular dystrophy pedigree. Squares and circles represent males and females, respectively. The fully shaded circle represents the female patient. The arrow indicates the proband. (b) Sanger sequencing validated the heterozygous c.1066C>T variant in the proband. (c) Sanger sequencing validated the heterozygous c.1102+2T>C variant in the proband. (d) Alignment of MFSD8 proteins in various organisms. The proline residue at position 356 of MFSD8 protein is highly conservative across seven different species. MFSD8, major facilitator superfamily domain containing 8.

Four members of a nonconsanguineous Chinese family with one individual affected by macular dystrophy were recruited for this study (Figure 1(a)). Ophthalmic exams, including visual acuity measurement and fundus examination, were performed on the proband (II:1). After obtaining written informed consent, peripheral venous blood samples of four members (I:2, II:1, II:2, and II:3) were collected. Genomic DNA was extracted from lymphocytes using a FlexGen Blood DNA Kit (CWBIO, Beijing, China) according to the manufacturer's instructions. Ethical permission was allowed by the Institutional Review Board of the Third Xiangya Hospital, Central South University, Changsha, Hunan, China. As samples from human subjects were involved, this study was carried out adhering to the tenets of the Declaration of Helsinki.

### 2.2. Whole Exome Sequencing and *In Silico* Analyses

Genomic DNA samples of peripheral venous blood from the proband (II:1) and her unaffected mother (I:2) were used for whole exome sequencing. In brief, DNA fragments were separately obtained with sonication. Exons were captured using the xGen Exome Research Panel v1.0 (Integrated DNA Technologies, Inc., Coralville, IA, USA) following the manufacturer's instructions to establish exome libraries. Whole exome sequencing on the Illumina NovoSeq 6000 sequencing system and *in silico* analyses were completed in the Chigene (Beijing) Translational Medical Research Center Co., Ltd. (Beijing, China). The general process was as follows. Clean reads were mapped onto the human reference genome (GRCh37/hg19) through the Burrows–Wheeler Aligner (BWA, http://bio-bwa.sourceforge.net/). Variants were called by the Genome Analysis Toolkit (GATK, https://software.broadinstitute.org/gatk/). SAMtools (http://samtools.sourceforge.net) and Pindel (http://gmt.genome.wustl.edu/packages/pindel/) were subjected to call single-nucleotide polymorphisms and insertions/deletions, respectively. Detected variants were annotated with the ANNOVAR (ANNOtate VARiation) tool (http://www.openbioinformatics.org/annovar/). Variants were filtered referring to public databases including the Single Nucleotide Polymorphism database v147, the 1000 Genomes Project, the Exome Aggregation Consortium, and the National Heart, Lung, and Blood Institute Exome Sequencing Project 6500 with minor allele frequency no less than 5%. The remained variants located in coding regions and canonical splice sites were taken for candidate identification. Several online programs including Protein Variation Effect Analyzer, Sorting Intolerant from Tolerant, Polymorphism Phenotyping v2, and MutationTaster were used to predict whether missense variants impacted protein functions [11, 12]. The effects of splicing variants were predicted by Human Splicing Finder and MaxEntScan online programs [13, 14]. Variants in genes associated with retinopathies were considered as potential disease-causing variants adhering to the standard variant interpretation guidelines of the American College of Medical Genetics and Genomics (ACMG) [15].

### 2.3. Sanger Sequencing

Variants screened by whole exome sequencing and suspected as potential causes were further validated in the proband and available family members. The polymerase chain reaction (PCR) amplification was performed with site-specific primers: 5′-TCCTGGTTATTTTTAGTGGAAAA-3′ and 5′-TTGGAGACTTCCAAAGACCAA-3′. Purified PCR products were directly sequenced using an ABI 3500 sequencer (Applied Biosystems, Foster City, CA, USA) adhering to manufacturer's instructions. The Chromas software (Technelysium Pty Ltd., Tewantin, QLD, Australia) was applied for analysis of sequence data.

### 2.4. Conservative Analysis

Multiple orthologous sequence alignments from seven different species were conducted by the online Clustal Omega tool (https://www.ebi.ac.uk/Tools/msa/clustalo/) as described previously [16].

## 3. Results

### 3.1. Clinical Manifestations

**Figure 2:**
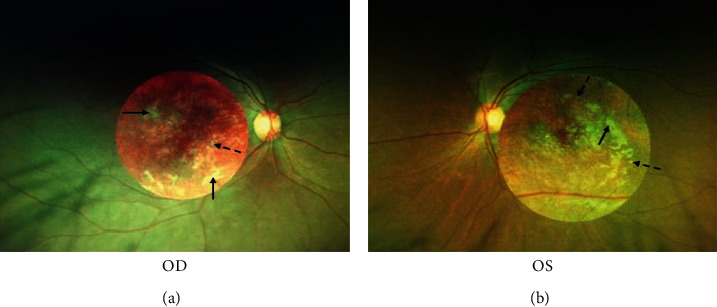
Fundus photographs of the proband with macular dystrophy. Fundus photographs showed macular degeneration with discrete (dashed arrow) and large confluent (solid arrow) macular drusen. OD, right eye; OS, left eye.

The proband presented with a two-year history of bilateral progressive visual loss and was diagnosed with macular dystrophy at 18 years of age. She had an uncorrected visual acuity of 16/200 in her right eye and 20/200 in her left eye. Fundus photographs showed macular degeneration with discrete and large confluent macular drusen (Figure 2). She had normal mental and motor abilities and denied histories of seizures and myoclonus.

### 3.2. Identification of *MFSD8* Gene Variants in the Proband

Whole exome sequencing and *in silico* analyses were performed on two members (the proband and her unaffected mother) of this family to seek the molecular etiology. Data with an average coverage of the target sequence no less than 20× accounted for 98.76% and 98.90%, respectively. Mean sequencing depth on target for the two members was 120.46× and 101.43×, respectively. A step-by-step bioinformatics pipeline containing base calling, low-quality read filtering, variant annotation, and biological function prediction was performed. In more than 270 known retinopathies genes (based on RetNet), only two novel variants, c.1066C>T and c.1102+2T>C, in the *MFSD8* gene (NG_008657.1 and NM_152778.4) were identified as being responsible for the macular dystrophy phenotype according to filtering steps of the methods. The compound heterozygous variants were further validated in the proband by Sanger sequencing (Figures 1(b) and 1(c)). The missense variant c.1066C>T (p.Pro356Ser) that was predicted by *in silico* analyses to probably damage the protein function was also detected in the proband's unaffected mother (I:2) and younger brother (II:3). Sequence alignment of multiple orthologous proteins revealed proline at position 356 of the MFSD8 protein was highly conservative in various organisms (Figure 1(d)). The splicing variant c.1102+2T>C, located in the intron 11 of the *MFSD8* gene, was not detected by Sanger sequencing in available unaffected members. It was predicted to probably affect splicing due to the alteration of the donor site. According to the ACMG guidelines for sequence variants, c.1066C>T and c.1102+2T>C variants were classified as “uncertain significance” and “pathogenic,” respectively. All of the above indicated that the compound heterozygous variants were likely to be the genetic cause of the proband's macular dystrophy phenotype.

## 4. Discussion

This study identified novel compound heterozygous variants, c.1066C>T and c.1102+2T>C, in the *MFSD8* gene as being possibly responsible for the family's macular dystrophy phenotype. In previous studies, the *MFSD8* gene mutations were the most common genetic cause of variant late-infantile neuronal ceroid lipofuscinosis (vLINCL), also termed as ceroid lipofuscinosis neuronal 7 disease (CLN7, OMIM 610951), which had an onset age of 1.5 to 5 years [17]. Symptoms of vLINCL included visual loss, seizures, psychomotor retardation, and premature death [18]. Some patients with vLINCL displayed visual impairment early, indicating that the retina is more sensitive to the *MFSD8* gene variant than the brain, and retinal neuron loss may precede brain neuron loss, supported by the findings in the *Mfsd8*-deficient mice [19]. Nevertheless, no hallmarks or severe neurologic symptoms were observed in the proband, supporting the *MFSD8* gene as a nonsyndromic macular dystrophy gene [20]. The findings were further underpinned by several recent reports of compound heterozygous or homozygous *MFSD8* gene variants identified in patients with nonsyndromic macular dystrophy, nonsyndromic retinitis pigmentosa, or more severe rod-cone dystrophy [8, 20, 21].

The MFSD8 protein, a ubiquitous lysosomal membrane protein, is a member of the major facilitator superfamily transporter proteins that transport small solutes through transmembrane ion gradients [6]. However, MFSD8 protein substrates are not clearly understood. In the retina, the MFSD8 protein is located in photoreceptor synaptic terminals and may be involved in the formation of synaptic vesicles [21]. However, the precise molecular mechanisms are unclear. Mutations in the *MFSD8* gene involved in a wide spectrum from hypomorphic to null alleles, and hypomorphic mutations could lead to partial function loss. According to the conjugation of these alleles, phenotypes are likely to vary from mild nonsyndromic retinopathies to severe syndromic retinopathies [1]. Intriguingly, the p.Glu336Gln variant, located proximately to p.Pro356Ser, was thought to be a ‘mild' variant giving rise to isolated macular dystrophy when combined *in trans* with either a null variant or a missense variant [21]. The p.Met454Thr homozygous variant was thought to be a ‘moderate' variant causing a more severe rod-cone dystrophy, but not vLINCL [21]. The p.Met454Thr variant, when paired with a more severe variant allele (p.Trp407Arg), may cause a severe neurological phenotype [22]. In the present study, the c.1066C>T (p.Pro356Ser) variant was predicted to be of “uncertain significance,” and it seemed to be a ‘mild' variant. The c.1102+2T>C variant was predicted to be “pathogenic,” and it probably affected splicing due to the alteration of the donor site. Similarly, a proximal c.1102G>C variant in the *MFSD8* gene was shown to result in a skipping of exon 11 and caused isolated macular dystrophy when paired with a c.1006G>C (p.Glu336Gln) variant [8]. These findings are consistent with the previously proposed genotype-phenotype correlation that the two severe allelic variants result in vLINCL, whereas milder variants on at least one allele cause nonsyndromic retinal degeneration diseases [20].

Some pathogenic *MFSD8* gene variants did not change the protein subcellular location in lysosomal compartments, suggesting that these variants probably altered protein stability or even functional property in lysosomes [7, 23]. The p.Pro356Ser variant locating in the transmembrane domain possibly alters protein stability along with functions rather than location of abnormal MFSD8 proteins in lysosomes. The c.1102+2T>C variant at the donor site was presumed to change the splicing pattern, and it thus most likely produced abnormal transcripts as well as truncated proteins. Further in-depth studies involving more functions are warranted. The flaw of this study was that the proband refused to perform optical coherence tomography, and the biomarkers such as the hyperreflective spot, the state of the ellipsoid zone, and the choroidal thickness could be considered as predictive factors for further evaluation of visual function and treatment response [24].

Taken together, this study identified novel compound heterozygous variants, c.1066C>T (p.Pro356Ser) and c.1102+2T>C, in the *MFSD8* gene. They are likely responsible for the isolated macular dystrophy phenotype in this family. Whole exome sequencing is likely to be an effective strategy for screening potentially causative variants of retinopathies with genetic heterogeneity and phenotypic variability. The results further broaden the *MFSD8* gene mutant spectrum and make possible more accurate genetic counseling for this family.

## Data Availability

The data used to support the findings of this study are included in the article.
